# Nano-engineered skin mesenchymal stem cells: potential vehicles for tumour-targeted quantum-dot delivery

**DOI:** 10.3762/bjnano.8.123

**Published:** 2017-06-07

**Authors:** Liga Saulite, Dominyka Dapkute, Karlis Pleiko, Ineta Popena, Simona Steponkiene, Ricardas Rotomskis, Una Riekstina

**Affiliations:** 1 Faculty of Medicine, University of Latvia, Raina blvd. 19, LV-1586, Riga, Latvia; 2 Biomedical Physics Laboratory, National Cancer Institute, P. Baublio Street 3b, LT-08406 Vilnius, Lithuania; 3 Life Science Center, Vilnius University, Sauletekio al. 7, LT-10257, Vilnius, Lithuania; 4 Laser research center, Vilnius University, Sauletekio al. 9, corp. 3, LT-10222, Vilnius, Lithuania

**Keywords:** endocytosis, mesenchymal stem cells, quantum dots, stem cell differentiation

## Abstract

Nanotechnology-based drug design offers new possibilities for the use of nanoparticles in imaging and targeted therapy of tumours. Due to their tumour-homing ability, nano-engineered mesenchymal stem cells (MSCs) could be utilized as vectors to deliver diagnostic and therapeutic nanoparticles into a tumour. In the present study, uptake and functional effects of carboxyl-coated quantum dots QD655 were studied in human skin MSCs. The effect of QD on MSCs was examined using a cell viability assay, Ki67 expression analysis, and tri-lineage differentiation assay. The optimal conditions for QD uptake in MSCs were determined using flow cytometry. The QD uptake route in MSCs was examined via fluorescence imaging using endocytosis inhibitors for the micropinocytosis, phagocytosis, lipid-raft, clathrin- and caveolin-dependent endocytosis pathways. These data showed that QDs were efficiently accumulated in the cytoplasm of MSCs after incubation for 6 h. The main uptake route of QDs in skin MSCs was clathrin-mediated endocytosis. QDs were mainly localized in early endosomes after 6 h as well as in late endosomes and lysosomes after 24 h. QDs in concentrations ranging from 0.5 to 64 nM had no effect on cell viability and proliferation. The expression of MSC markers, CD73 and CD90, and hematopoietic markers, CD34 and CD45, as well as the ability to differentiate into adipocytes, chondrocytes, and osteocytes, were not altered in the presence of QDs. We observed a decrease in the QD signal from labelled MSCs over time that could partly reflect QD excretion. Altogether, these data suggest that QD-labelled MSCs could be used for targeted drug delivery studies.

## Introduction

Despite remarkable advances in targeted therapies of various human malignancies, cancer is one of the leading causes of death worldwide [1]. Nanoparticles (NPs) could be linked to various drugs, thereby making them suitable for tumour imaging and targeted therapy [2]. However, the fact that NPs are quickly recognised by immune cells and cleared from the blood stream by reticuloendothelial system limits their utility as drug carriers [3]. Recent studies have shown that nano-engineered mesenchymal stem cells (MSCs) could be used as tumour-targeted therapeutic carriers, reflecting their tumour-homing capabilities [4–6].

MSCs are present in many tissues of the human body, including bone marrow, adipose tissues, skin and dental pulp. According to current understanding, MSCs are defined as adherent cells with a spindle-like morphology, expressing CD105 (SH2 or endoglin), CD73 (SH3 and SH4), CD106 (VCAM-1), CD44 (hyaluronic acid receptor), CD90 (Thy 1.1), CD29, CD146 and CD166 surface markers [7–8]. MSCs can be induced to differentiate in vitro into adipogenic, osteogenic, chondrogenic and myogenic cells. Moreover, other cell types, such as neurons, glial cells and smooth muscle cells, could be obtained from MSCs under the appropriate cell culture conditions [9–10].

Among the broad variety of investigated NPs, quantum dots (QDs) have demonstrated extensive application capabilities. High photostability and brightness, broad excitation and narrow fluorescence-emission spectra are some of the main properties required for the generation of new fluorescent nano-agents. The unique optical and electronic properties of QDs indicate their great potential in cancer diagnostics. The photoluminescence spectrum of carboxyl QD655 makes them ideal candidates for cancer theranostics as it overlaps with the optical transparency window of biological tissue [11]. Additionally, large and easily altered surfaces facilitate modifications of various NPs. These modifications increase the solubility of QDs to make QDs unnoticeable by the immune system, increase the QD half-life in the blood stream and target QDs to specific ligands or antigens [12]. Different therapeutic and recognition molecules can be attached to the surfaces of NPs and act synergistically [13–14]. QDs were also chosen for their applicability as resonant energy donors in photodynamic therapy. For example, the second-generation photosensitizer chlorin e6 has the absorption band at 654 nm and carboxyl QD655 would be excellent energy donors in such complexes. There were successful attempts to use a similar quantum dot–chlorin e6 complex in photodynamic cancer therapy [15]. Another study has shown that QDs, conjugated with antibodies against CD44, a marker of cancer stem-like cells, can be selectively engulfed by breast cancer cells [16]. Such surface modifications increase the potential of QDs for the use in targeted cancer diagnostics and therapies.

There is still doubt regarding the potential harmful effects of NPs or QDs on the differentiation capacity and self-renewal ability of adult stem cells. CdSe/ZnS QD labelling has been reported to adversely affect the osteogenesis and chondrogenesis capacities of bone marrow MSCs [17]. The impact of QD labelling on the biological properties of targeted stem cells, such as proliferation, cell cycle, and apoptosis, remains elusive. Therefore, further research on MSCs with regard to the delivery of QDs for monitoring and treating tumours is required.

Skin is the largest organ of the human body. It ensures the protection and insulation of the inner tissues [18] and also acts as a barrier against the penetration of QDs [19]. Nano-engineered skin MSCs could be used in cell-based skin cancer (SC) therapies [20–21]. MSCs loaded with anti-cancer drugs can reduce melanoma tumour growth in vivo, suggesting that these molecules are suitable vectors for therapeutic applications [22].

The aim of the present study was to analyse the accumulation, release, toxicity and functional effects of carboxyl QD655 on skin-derived MSCs to assess their potential use as vectors for the targeting of SC or other tumours.

## Results

### Optimal QD labelling conditions for MSCs

**Figure 1:**
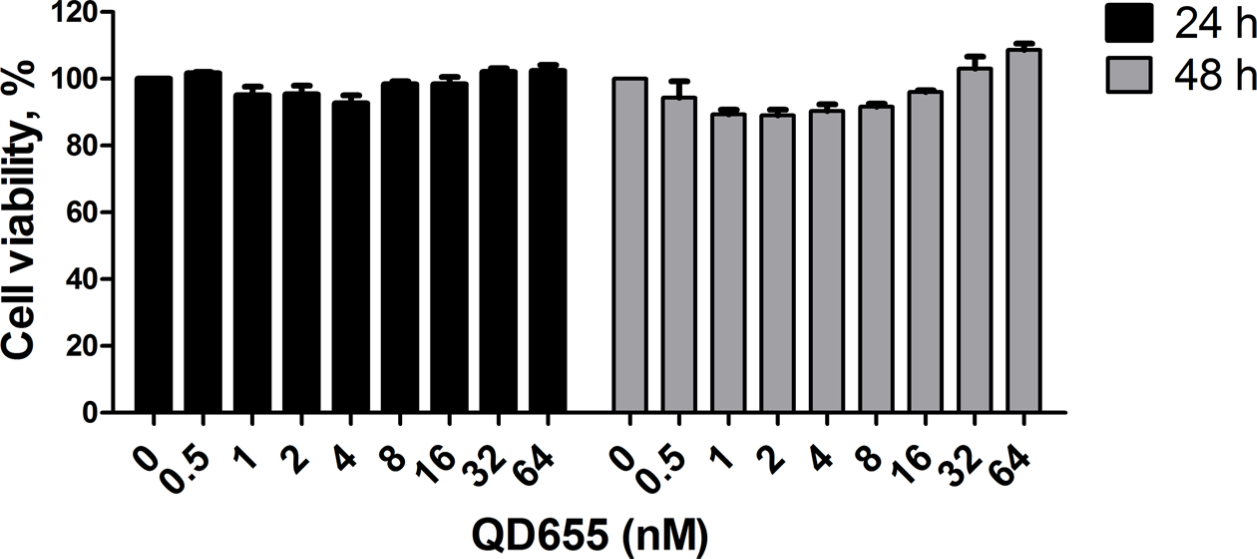
The concentration-dependent effect of QDs on the viability of MSCs. Viability was measured by a colorimetric assay (CCK-8) after incubation with QDs at 0.5–64 nM for 24 and 48 h.

The concentration-dependent cytotoxicity of QDs was analysed in MSC cultures after 24 and 48 h using a colorimetric CCK-8 assay, which measures intracellular dehydrogenase activity (Figure 1). QDs did not significantly affect MSC viability after 24 or 48 h at any of the tested QD concentrations.

**Figure 2:**
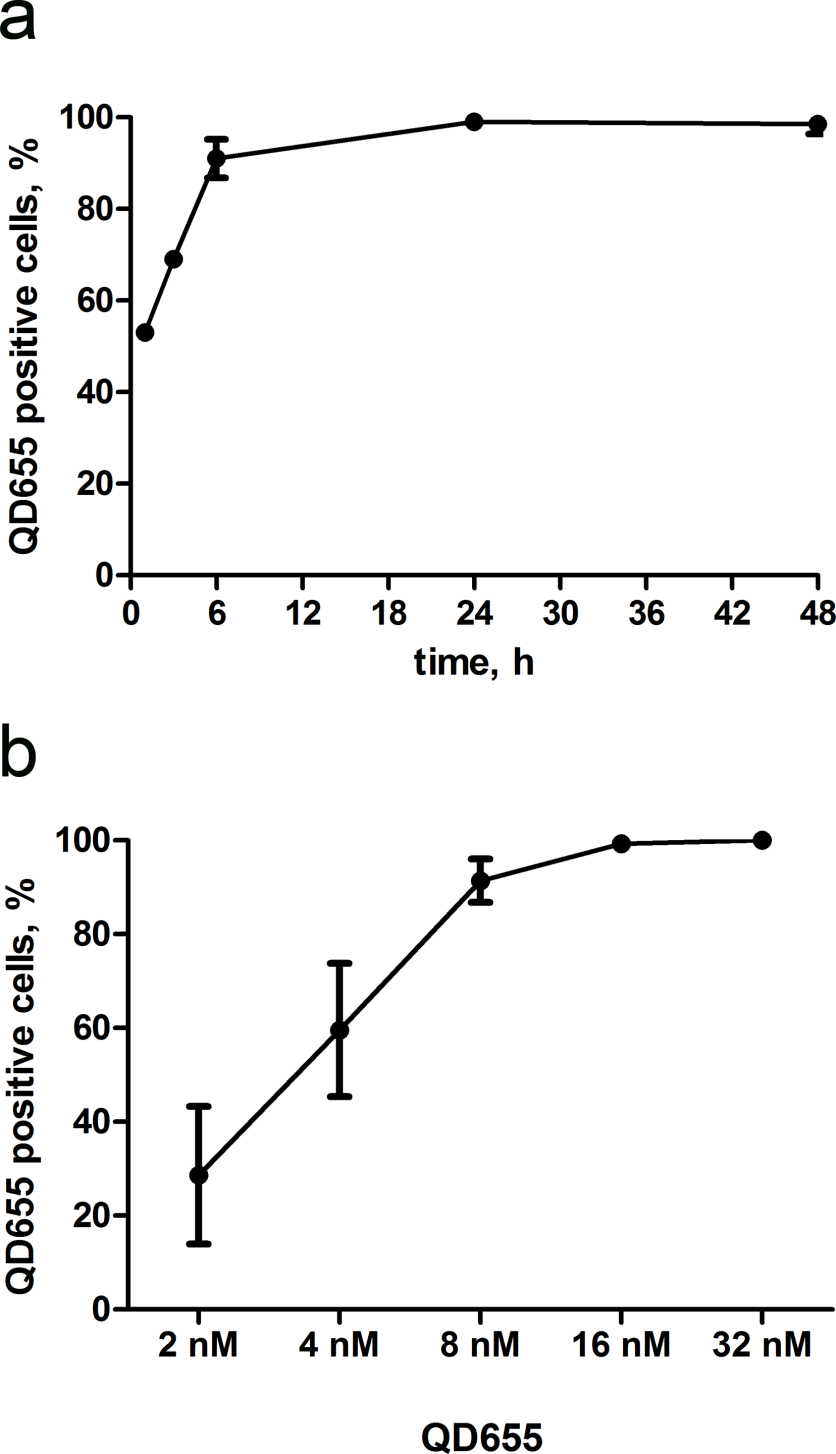
Evaluation of the optimal QD uptake conditions in skin MSCs. Time-dependent (a) and concentration-dependent (b) uptake dynamics in MSCs using flow cytometry analysis. The percentage of QD655-positive cells was obtained from the analysis of flow-cytometry histogram data.

In order to select the optimal incubation time for QD uptake in skin MSCs, cells were incubated with 16 nM QDs for time periods ranging from 15 min to 48 h (Figure 2a). The QD uptake kinetics was calculated based on changes in fluorescence intensity. The plateau phase was reached after 24 h of incubation, consistent with observations in other cell lines [23]. The optimal incubation time for QD uptake was 6 h, after which up to 95% of the cells had incorporated QDs. Thus, a 6 h incubation time was used in all experiments, unless otherwise stated.

The optimal QD concentration for the uptake experiments was determined using serial dilutions of QDs from 2 up to 32 nM (Figure 2, b). The QD-positive cell number exponentially increased, and saturation was obtained at 16 nM, when cells were 99% QD-positive. Therefore, a 16 nM QD concentration was selected for further experiments, unless otherwise stated.

**Figure 3:**
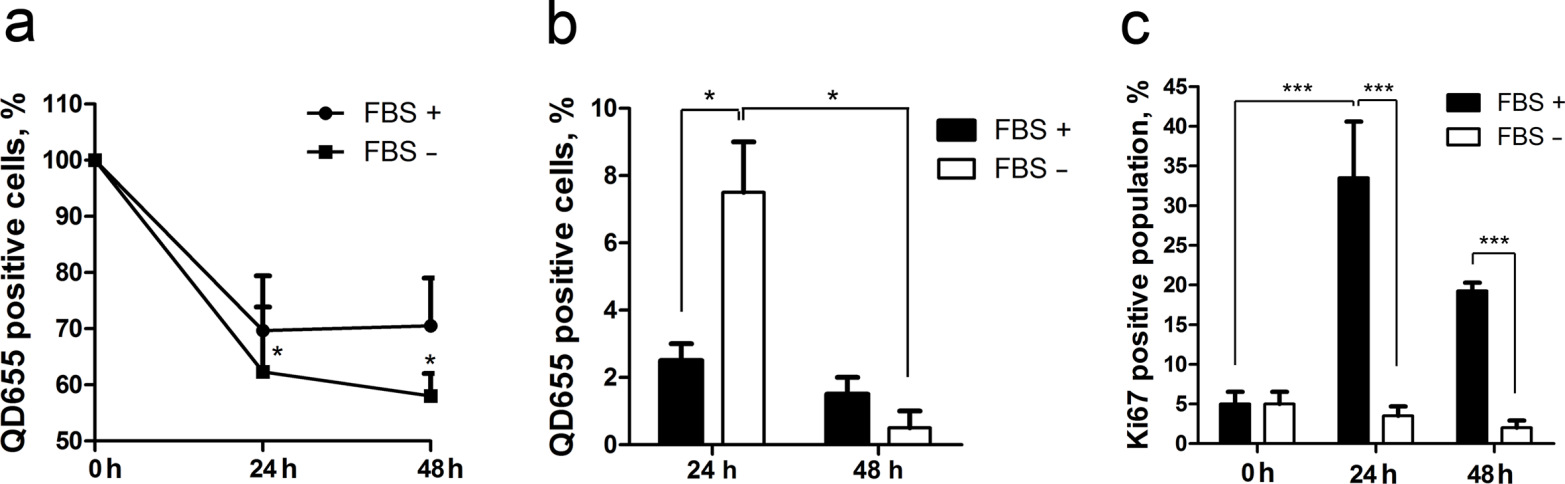
The release of QDs from MSCs. (a) QD loss in complete medium (FBS +) and serum-free medium (FBS −) after primary QD labelling (0 h) and 24 and 48 h after labelling. The statistical significance is shown in comparison to 0 h. (b) Secondary QD labelling of MSCs by supernatants from primarily labelled MSCs. (c) The comparison of Ki67 expression in complete and serum-free medium after labelling (0 h) and after 24 h and 48 h of cultivation; *p-value < 0.05, ***p-value < 0.001.

To determine whether MSCs release QDs in the environment after uptake, the supernatant was removed from cells after primary QD labelling. After rigorous rinsing, fresh complete or serum-free medium was applied to the QD-labelled cells. Next, the QD fluorescence intensity was determined in cells at 24 and 48 h after primary labelling. We observed a 30% decrease of the QD signal in cells propagated in complete medium and a 40% decrease of the QD signal under serum-free conditions after 24 h of incubation (Figure 3a). After 48 h, the number of QD-positive cells decreased even further in serum-free cultivated cells (Figure 3a). Supernatant from primarily QD-labelled MSCs was transferred to fresh MSCs for secondary labelling experiments. After 24 h, 3% of the cells in complete medium had taken up QDs, whereas under serum-free conditions, 7% of MSCs had taken up QDs in the secondary labelling experiments (Figure 3b). After 48 h QD uptake was detectable in approximately 1.5% of cells cultivated either in complete or serum-free medium (Figure 3b).

To determine the effect of cell division on the decrease of the QD signal, QD-labelled MSCs were propagated in complete and serum-free medium. Ki67 expression was clearly inhibited in cells cultivated in serum-free medium, which did not proliferate after 24 and 48 h, thereby excluding the probability of QD transfer to daughter cells (Figure 3c). Inhibition of proliferation was additionally confirmed by analysing the cell number in the respective medium (data not shown). The addition of QDs did not change the expression of Ki67 (data not shown).

### QD effect on immunophenotype, proliferation and differentiation of MSCs

**Figure 4:**
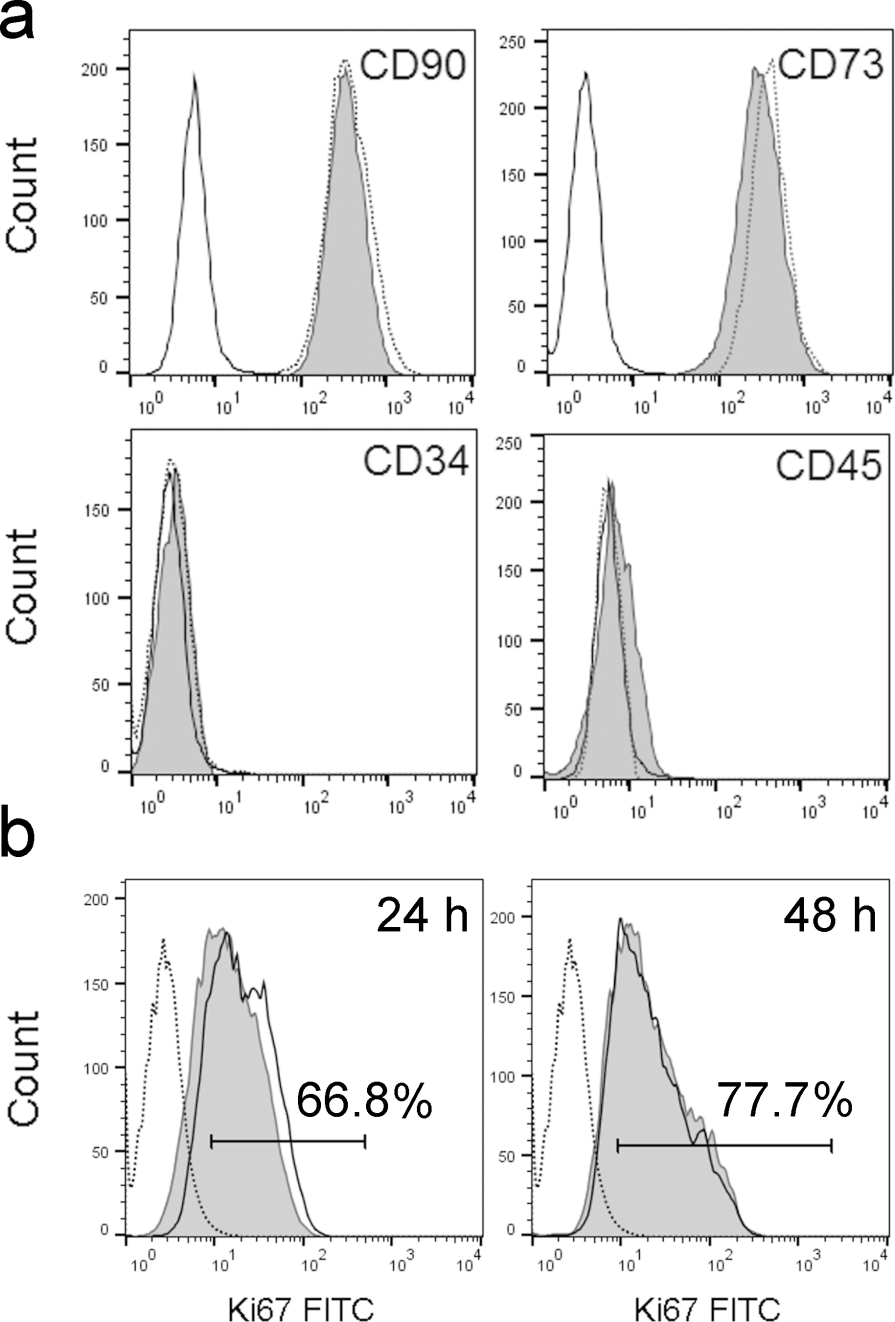
Representative data on the impact of QDs on immunophenotype and proliferation of MSCs. (a) Characterization of MSC markers CD90, CD73 and hematopoietic markers CD34 and CD45 in MSCs. Open histogram: unlabelled cells, dotted-line histogram: MSCs without QDs, grey histogram: QD-labelled MSCs. (b) Ki67 expression in MSCs after 24 h and 48 h of incubation with 16 nM QDs. Open histogram: unlabelled cells, dotted-line histogram: isotype control, grey histogram: QD-labelled cells.

The skin MSC population used in the present study was over 95% positive for MSC markers CD73 and CD90, whereas hematopoietic markers CD45 and CD34 were not expressed (Figure 4a). To estimate the effect of QDs on the MSC immunophenotype, expression of CD73 and CD90 was analysed after incubation with QDs for 48 h. Although CD105 is often used as a MSC marker together with CD73 and CD90, this marker was excluded from the analysis because of the fluorescence channel overlap with QDs (APC label, FL4). The data showed that QDs did not change the expression of CD73, CD90, CD34 and CD45 in MSCs (Figure 4a).

The effect of QDs on proliferation was analysed based on Ki67 expression (Figure 4b). After incubation for 24 h, 67% of unlabelled and QD-labelled MSCs expressed the Ki67 marker. After 48 h, the Ki67-positive population increased to 78% in both cell populations. QDs did not show any effect on the proliferation of MSCs.

**Figure 5:**
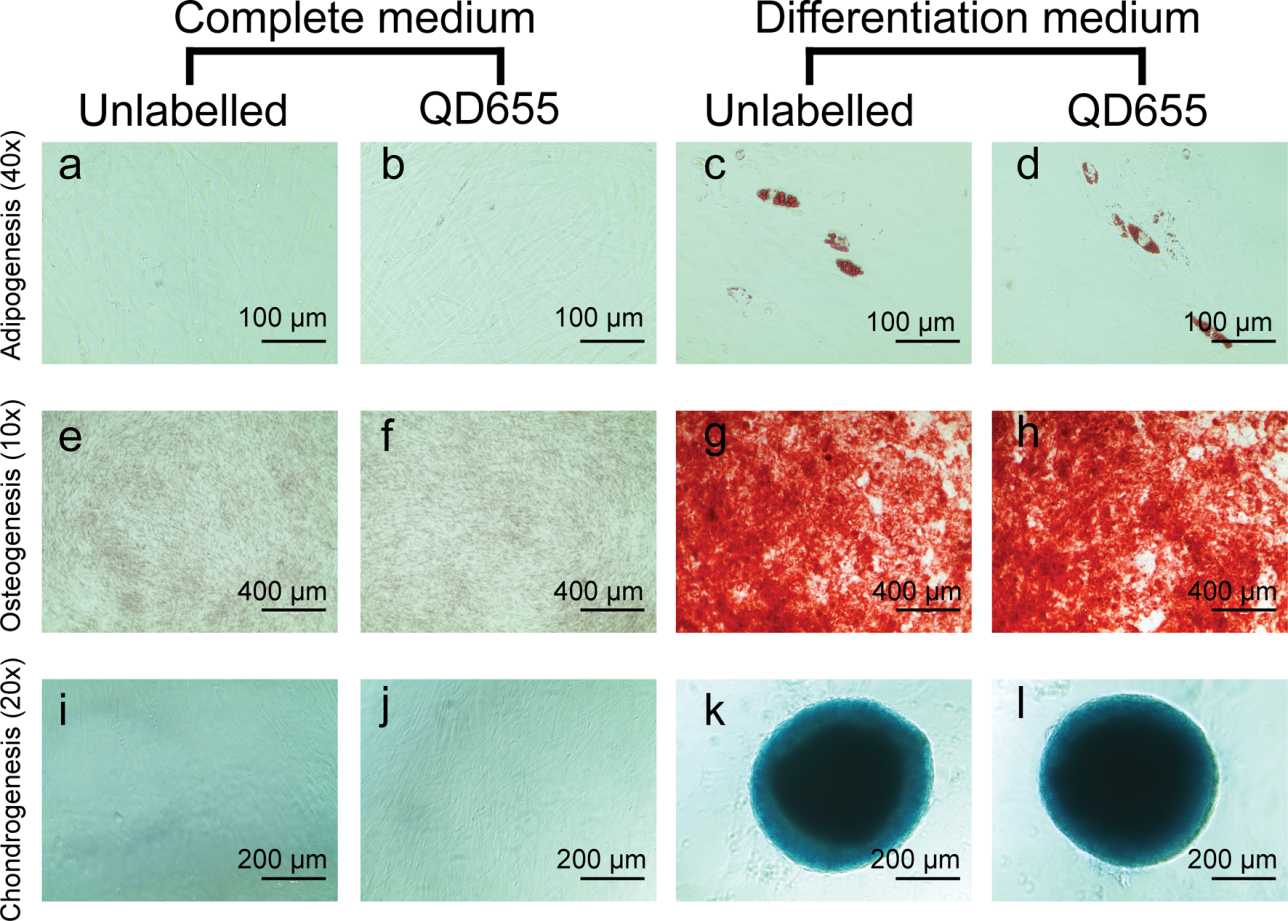
Differentiation of MSCs into adipocytes, chondrocytes and osteocytes. Oil Red O staining of cells in complete medium without (a) or with QD labelling (b); cells in adipogenesis medium without (c) or with QD labelling (d). Alizarin Red S staining on cells in complete medium in the absence or presence of QDs (e, f); cells in osteogenesis differentiation medium in the absence or presence of QDs (g, h). Alcian Blue staining on cells in complete medium (i, j) and chondrogenesis differentiation medium (k, l) in the absence or presence of QDs.

**Figure 6:**
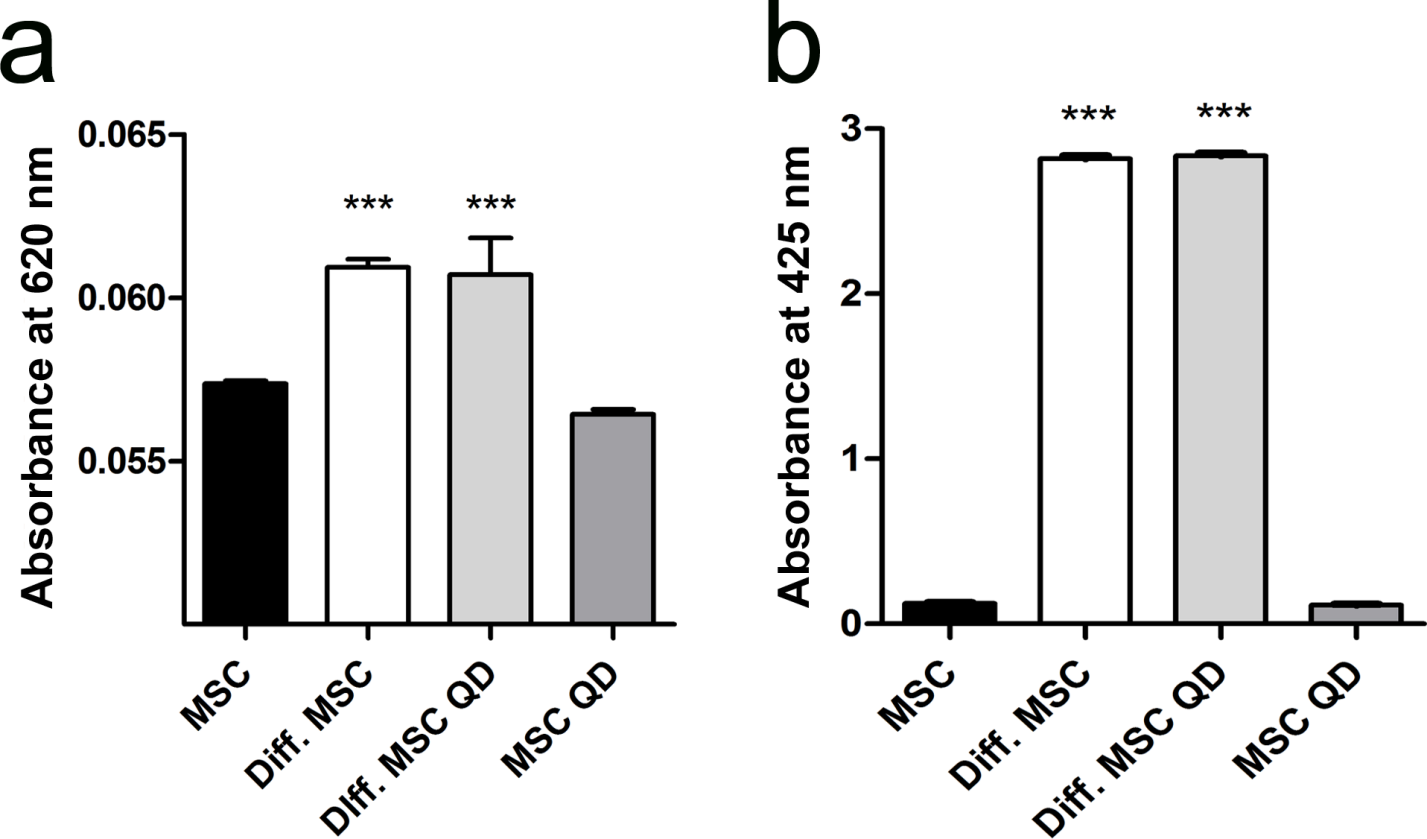
Quantification of osteogenesis and chondrogenesis in MSCs. Absorbance of Alizarin Red S (a) and Alcian Blue (b) extraction from MSC differentiation. Diff. MSC: differentiated MSCs, diff. MSC QD: differentiated MSCs labelled with QDs. Significance compared between differentiated and undifferentiated samples; ***p-value < 0.001.

The differentiation of MSCs into adipocytes, chondrocytes and osteocytes was not affected by the presence of QDs (Figure 5). Quantification assays for Alcian Blue staining and Alizarin Red S staining confirmed that QDs did not influence chondrogenesis and osteogenesis of skin MSCs (Figure 6).

### Analysis of the uptake pathway of QDs

**Figure 7:**
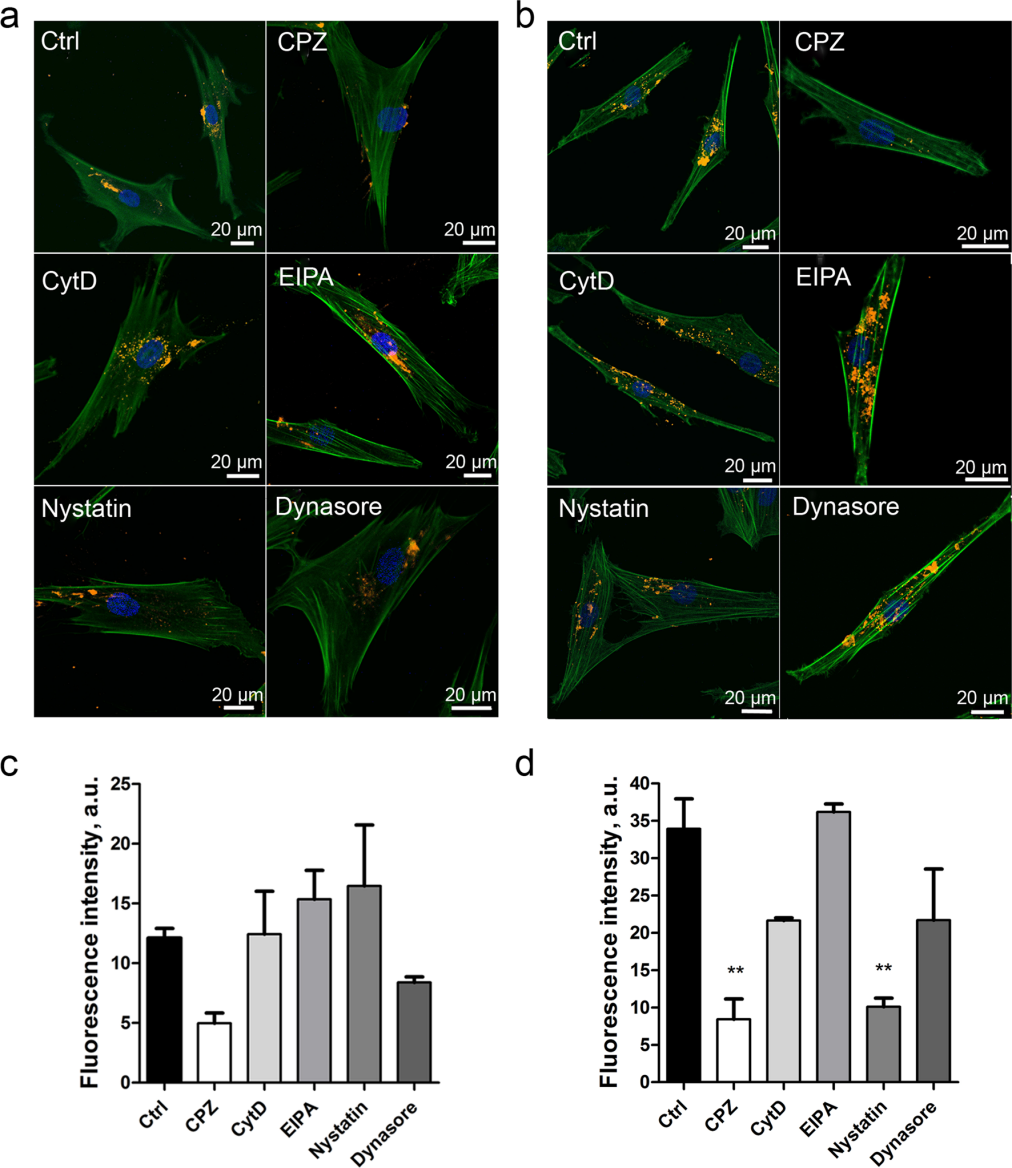
QD endocytic pathway in MSCs. QD uptake pathway in MSCs labelled with QDs in complete medium (a) or in serum-free medium (b). Uptake pathways were blocked using the endocytosis inhibitors CPZ, CytD, EIPA, nystatin and dynasore. Three overlaid channels represent Hoechst (blue), Phalloidin Alexa Fluor488 (green), carboxyl QD655 (yellow). Representative data are shown. QD fluorescence signal was quantified in complete (c) and in serum-free medium (d) cultivated MSCs. Statistical significance shown for the respective sample in comparison to control (Ctrl) sample; **p-value < 0.01.

MSCs were pre-treated with endocytosis inhibitors and subsequently labelled with QDs. The effect of serum proteins on the efficiency of QD uptake was analysed based on the comparison of QD uptake in complete and serum-free media (Figure 7). The effect of endocytosis inhibitors differed between complete and serum-free medium. In complete medium, a tendency of decreased QD uptake was observed using chlorpromazine (CPZ), an inhibitor of clathrin-mediated endocytosis (Figure 7a, c). In serum-free medium, QD uptake was significantly inhibited by CPZ and nystatin, an inhibitor of caveolin/lipid raft-mediated endocytosis (Figure 7b,d). In serum-free medium, the cells internalized more QDs according to the fluorescence intensity analysis (Figure 7c,d).

**Figure 8:**
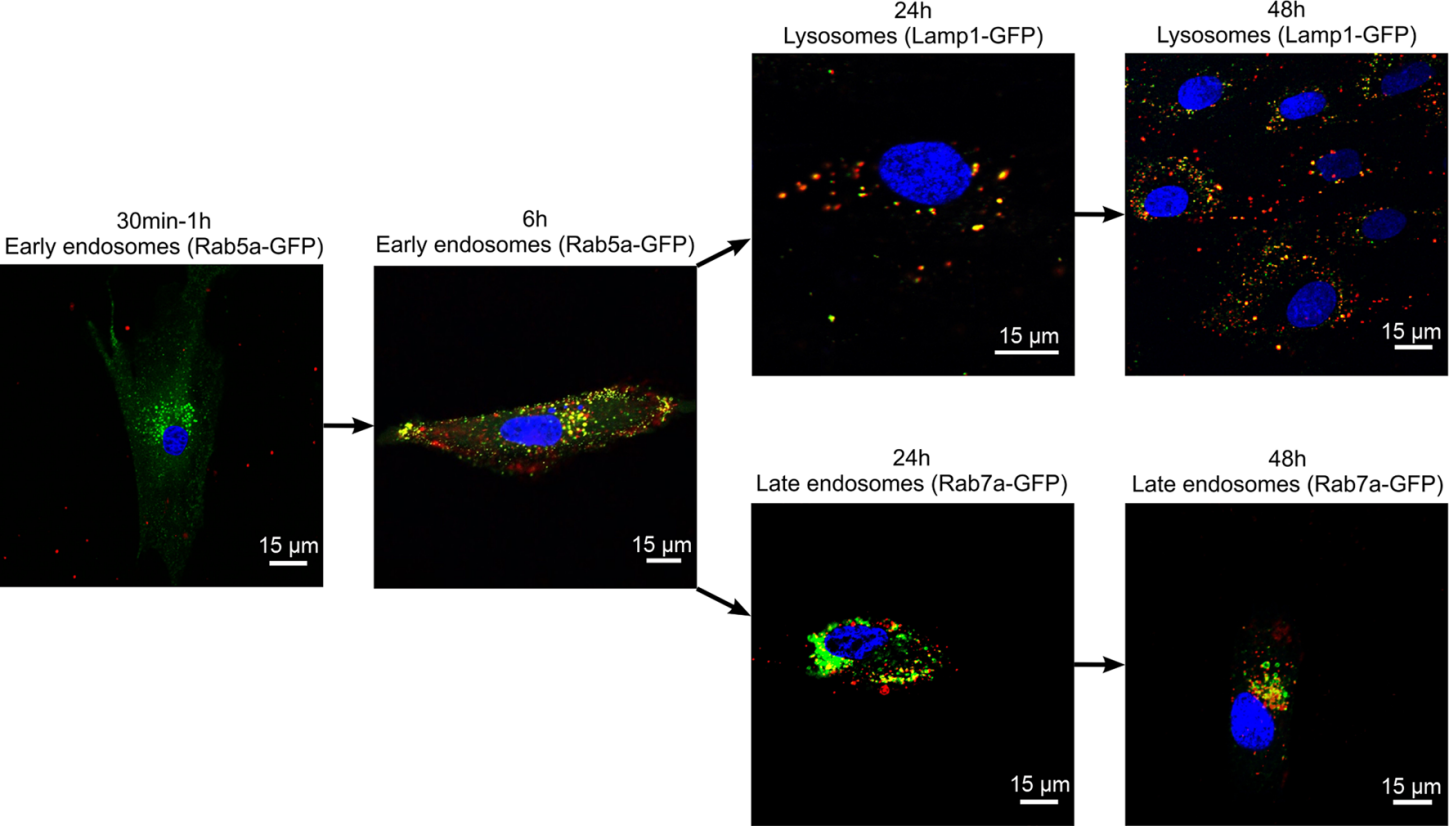
QD co-localization with endosomal compartments. Three overlaid channels represent the nucleus (blue), carboxyl QD655 (red) and Rab5a-GFP (early endosomes), Lamp1-GFP (lysosomes) or Rab7a-GFP (late endosomes) (green). Yellow colour demonstrates co-localization. Representative data are shown.

The intracellular localization of QDs after uptake was observed in BacMam 2.0-transfected MSCs. Excessive QD accumulation was initiated between 1 and 6 h. After 6 h, most of the QDs were localized in early endosomes (Figure 8) in both the cell periphery and perinuclear area. After 6 h, almost no QDs were localized in mature endosomes (data not shown). After 24 and 48 h, QD-containing early endosomes matured into late endosomes and lysosomes.

## Discussion

Human MSCs have been widely investigated for their potential use in various therapeutic applications, due to their plasticity and migration ability. It has been proposed that MSC migration towards injury and inflammation sites could be used to deliver diagnostic and therapeutic nano-agents [24]. Studies on melanoma [25], prostate cancer [26], breast cancer [6] and lung cancer [27] have shown the ability of MSCs to home to cancer sites in vivo. In the tumour microenvironment, MSCs play a role in the formation of the tumour stroma and support cancer metastasis [28]. Lourenco et al. showed that MSC migration towards cancer cells is induced by MIF–CXCR4 chemotaxis [29]. Moreover, in close proximity of the tumour, cancer-associated fibroblast formation is induced by the release of vesicles containing miRNA from cancer cells. This leads to melanoma growth and invasion [30]. Therefore, skin-derived MSCs could serve as an appropriate model to study the stem cell (SC) tumour microenvironment and design SC-targeted therapeutics. In the present study, we addressed whether QD-loaded skin MSCs could serve as vectors to deliver NPs to cancer sites. To answer this question, the biological response of skin MSCs to QDs was investigated.

The results showed that QDs do not induce changes in immunophenotype, proliferation and viability of skin MSCs, indicating that QDs are biocompatible with MSCs. These results are consistent with those of studies on bone marrow mesenchymal stem cells and mouse embryonic stem cells, which show similar effects after QD labelling [31–32]. We observed variations in Ki67 expression in skin MSCs, regardless of QD addition, which might reflect the differences in donor age and passage number [33]. In the present study, we observed that QD labelling did not interfere with skin MSC differentiation into osteocytes, chondrocytes and adipocytes, and moreover, QDs did not induce spontaneous differentiation. Similarly, Shah et al. reported that carboxyl QDs do not alter the differentiation potential of human bone marrow stem cells [31]. Thus, QD-labelled MSCs are potentially safe to use in long-term tumour imaging and cell tracking experiments. Although there is a great deal of concern about the potential hazards of QDs containing heavy metals, the toxicity of QDs is a topic of controversy. The toxicity and ecotoxicity of QDs is studied at various levels of biological organization, from cell monolayers to primates and even ecosystems [34–35]. The potential toxicological effects of QDs are usually based on the release of free cadmium (Cd) [36]. However, QD shell and surface coatings protect the core, which contains toxic inorganic semiconductor materials. Unless coatings are damaged, QDs are mainly non-toxic [37]. Recently, Yaghini et al., by using non-photolytic visible wavelength excitation, have shown the formation of superoxide anion radicals by photoexcited CdSe/ZnS QDs [38]. Thus, the QDs may induce phototoxic reactions in labelled cells, which could be a desirable event in targeted tumour therapy.

The optimal uptake conditions for NPs could depend on the particle size, surface modifications, protein corona, and recipient cell line. Previous studies have suggested the incubation of NIH3T3 mouse fibroblasts with 16 nM QDs for 6 h as the optimal conditions for cellular uptake experiments [39]. Given the lack of standardized NP uptake conditions in MSCs, we adjusted the protocol for QD uptake in human skin MSCs. The results showed that a 6 h incubation with 8 or 16 nM QDs is optimal for QD accumulation in more than 95% of the MSC population (Figure 2). Notably, a 1 h incubation with 5 nM and 20 nM QDs has previously been reported as sufficient for the labelling of rat bone marrow MSCs [40]. However, optimization of the NP incubation time and concentration is necessary in each individual experimental setting.

The uptake pathway of NPs varies depending on the cell and particle type. One of the factors affecting uptake is the protein corona that forms around NPs in serum-containing medium. Protein aggregates decrease gold NP uptake depending on size and cell type [41]. In the present study, we analysed the QD uptake pathways under both serum-containing and serum-free conditions. Selected inhibitors for the major uptake pathways were applied to cells prior to QD incubation. In serum-containing medium, decreased QD uptake in MSCs was observed after treatment with CPZ (Figure 7a,c). CPZ is an inhibitor of clathrin-mediated endocytosis through the anchoring of the clathrin and adaptor protein 2 (AP2) complex to endosomes, thereby preventing the assembly of coated pits at the inner plasma membrane [42]. In serum-free medium, QD uptake was decreased by CPZ and nystatin (Figure 7b,d). nystatin is an inhibitor of caveolin/lipid raft-mediated endocytosis, which disassembles caveloae and cholesterol in the membrane, but does not interfere with clathrin-mediated endocytosis [43]. Zhang et al. and Xiao et al. showed that dendritic cells and breast epithelial cells uptake carboxyl QDs via the clathrin-mediated pathway [44–45]. By contrast, experiments in HEK cells showed the uptake of carboxyl QDs through caveolin/lipid raft-mediated endocytosis; although it has been reported that caveolin-mediated endocytosis is the dominating uptake route in endothelial cells, smooth muscle cells and adipocytes [44,46]. Damalakiene et al. demonstrated that QDs possessing a protein corona are differently recognized by NIH3T3 cells and internalized by different pathways [23], consistent with the data from the present study. Interestingly, MSCs showed more effective internalization of QDs under serum-free conditions, as the protein corona interferes with QD uptake in skin MSCs. The composition of the protein corona could either enhance or decrease the cellular uptake of polystyrene-based NPs, depending on nanoparticle functionalization [47]. We have showed that NP uptake in skin MSCs is an active process and does not occur passively. For the development of cell-based tumour-targeted therapies, elucidation of the endocytic pathway is very important, because it may have an effect on the fate of QDs and/or QD-linked drugs within the cell. For example, after QD uptake by clathrin-mediated endocytosis, the QDs subsequently could be transferred to lysosomes for degradation or, depending on their surface coating, recycled to the cell surface [48]. On the contrary, QDs taken up by caveolae-dependent endocytosis could bypass lysosomes and avoid lysosomal degradation [48]. Taken together, the accumulated experimental evidence suggests that the QD uptake pathway depends on the cell type, the formation of a protein corona and added functional groups on the NPs.

Intracellular localization of QDs in endosomes and lysosomes has been reported to be a common pathway following NP uptake through which particles are brought for lysosomal degradation [46,49]. We observed internalization of QDs in early endosomes after 6 h of incubation, followed by re-localization to late endosomes/lysosomes after 24 and 48 h of incubation (Figure 8). Cell division, excretion and degradation are the main mechanisms reported for QD signal elimination over time [50–51]. It has been implicated that the elimination rate depends on the particle size. Smaller NPs lead to faster elimination [50–52]. In the present study, we observed that the transfer of QDs to daughter cells during cell division is not the main mechanism involved in QD signal reduction in skin MSCs. Similar observations have been reported in the study of mouse embryonic stem cells, where QD loss was still detected after the inhibition of cell proliferation, suggesting that QDs might be excreted from cells [50]. Indeed, we demonstrated that MSCs could be repetitively labelled by the removal of supernatants from QD-loaded MSCs, confirming the presence of released QDs in the supernatant. After secondary labelling, the number of QD-positive MSCs was two times higher in serum-free medium compared to complete medium, likely indicating that the protein corona interferes with the QD uptake. Many types of stem cells have membrane transporters for the elimination of toxic reagents [53]. The induction of ABC transporter P-glycoprotein increases the elimination of QDs from HEK and HepG2 cells, while its inhibition demonstrated an opposite effect. The elimination rate was higher in HEK cells, because of the stem cell phenotype [54]. Expression of P-glycoprotein has also been reported in MSCs [4]. However, other data in mouse embryonic and kidney stem cells indicate that QD depletion likely occurs during cell division and that no excretion mechanisms could be observed [32]. Taken together, these data indicate that QD elimination mechanisms may be cell-type dependent. The results from skin MSCs demonstrated that the depletion of the QD signal over time could be explained by QD degradation and excretion. The fact that NPs are released from MSCs is important because of the intended use of MSCs as NP delivery vectors. We propose that cancer cell and MSC co-culture model could be used to demonstrate the applicability of QD-labelled MSCs for cancer theranostics. For example, Pietila et al. have demonstrated that direct cell–cell contact is required for QD–mortalin antibody transfer from nano-engineered MSCs to the breast cancer cell line MDA-MB-231 in vitro [55]. Alternatively, QDs or MSCs loaded with QD–drug conjugates could be used in melanoma xenograft models in vivo as was shown in a study by Studeny et al. where IFN-β-MSCs co-injected with a human melanoma cell line suppressed tumour growth in nude mice [25].

Altogether, we propose several reasons why QD-labelled skin MSCs could serve as a promising NP delivery vector. First, QD labelling would enable MSC tracking and visualization of the tumour microenvironment. Next, the cells in the tumour would take up the released QDs and then the formation of ROS could be induced through photoactivation, leading to cancer cell apoptosis. Last but not least, the secretion of sTNFR1 by skin MSCs could downregulate the pro-tumourigenic inflammatory responses [56–58].

## Conclusion

Herein, we showed that carboxyl-coated QDs are biocompatible with skin MSCs. The proliferation, immunophenotype and differentiation potential of MSCs was not affected by QD accumulation in the cells. In the presence of serum, QDs were internalized in MSCs through clathrin-mediated endocytosis, whereas in the absence of serum, QD uptake occurs through the clathrin and caveolin/lipid raft-mediated endocytosis pathways. The loss of QD signal over time may possibly be explained by the excretion of QDs from MSCs, which could favour the use of MSCs as drug delivery vectors. These data validate the potential use of skin MSCs as NP delivery vectors for tumour-targeted therapies.

## Experimental

### Mesenchymal stem cell culture

Human skin samples were obtained from post-surgery materials with authorized approval from Research Ethics Committee, Institute of Experimental and Clinical Medicine, University of Latvia (issued 04.06.2014). Dermal MSC cultures were obtained as described elsewhere [59]. In brief, skin specimens were washed with cold phosphate-buffered saline (PBS), cut into 4–6 mm^2^ pieces and incubated in 0.6 U/mL dispase (Roche, Switzerland) for 1–3 h at 37 °C to remove the epidermis. Dermis was minced manually before enzymatic digestion with 0.62 Wunsch U/mL Liberase Blendzyme 1 (Roche, Switzerland) for 30 min at 37 °C, then dissociated by vigorous pipetting and passed through a 70 μm cell strainer, followed by centrifugation at 400*g* for 5 min. The pellets were suspended in cultivation medium containing DMEM/F12 (3:1 v/v) supplemented with 10% of FBS and antibiotics (100 U/mL penicillin, 100 μg/mL streptomycin) (all from Sigma-Aldrich, USA). Cell suspensions were transferred into 25 cm^2^ tissue culture flasks and grown until reaching 80% confluence in a humidified chamber at 37 °C with 5% CO_2_. Cells were trypsinized with 0.25% trypsin–EDTA solution (Sigma-Aldrich, USA). Cells at passages 2 to 5 were then frozen at −80 °C for long-term storage in a cell bank. All experiments were performed in compliance with the relevant laws and institutional guidelines. In this study five independent donor skin MSC cultures from passage 4 to passage 8 were used.

### MSC surface marker analysis

Phenotyping of cell surface markers was performed by flow cytometry. The cells were stained with CD34-PE and CD45-FITC (all from BD Biosciences, USA), CD90-FITC (Dako, USA), CD73 PE (Abcam, USA) and isotype controls IgG1-FITC (Dako, USA), IgG1-PE (BD Biosciences, USA), and IgG2A-APC (BD Biosciences, USA). Flow cytometry data were acquired using a Guava EasyCyte 8HT flow cytometer and analysed using ExpressPro software (Merck Millipore, USA) comparing unlabelled, marker-labelled and isotype control populations in FL-1, FL-2 and FL-4 channels.

### Quantum dots

Qdot^®^ 655 ITK™ non-targeted carboxyl-coated quantum dots were purchased from Thermo Fisher Scientific, USA. QDs are composed of a CdSe core with a ZnS shell that are coated with amphiphilic polymers and functionalized with carboxylate. The QDs have an emission maximum at 655 nm. Xu et al. measured the hydrodynamic diameter of the nanoparticles to be 14.55 ± 4.157 nm and a zeta potential of −35.1 mV [60]. The stock solution is 8 µM in 50 mM borate, pH 9.0. Further preparations of the QD solution are described in each methodological part separately.

### QD uptake dynamics using flow cytometry

To estimate the optimal QD concentration for uptake experiments, MSCs were seeded at a density of 5 × 10^4^ cells per well in a 12-well tissue culture polystyrene plate and labelled with QDs at various concentrations in the range of 0.5 to 64 nM for 6 h in complete or serum-free medium. To determine the accumulation dynamics, 8 nM or 16 nM QDs were applied to MSCs and incubated for 0.5, 1, 3, 6, 24 and 48 h in complete medium. The cells were subsequently harvested by trypsinization, centrifuged at 250*g* for 5 min and resuspended in 200 μL of PBS. The samples were acquired on a Guava EasyCyte 8HT flow cytometer and analysed using ExpressPro software (Merck Millipore, USA) in channel FL4, comparing unlabelled and labelled cell populations.

### Cell-viability assay

The impact of carboxyl-coated QD655 on the viability of MSCs was analysed using the Cell Counting Kit 8 (CCK-8) (Sigma-Aldrich, USA**)**. A total of 5 × 10^3^ cells per well were seeded onto 96-well plates in 100 μL of complete medium. The next day, QDs were added in serial dilutions at a twofold dilution in complete medium. The range of the tested QD concentrations ranged from 0.5–64 nM with twofold dilution. The cells were incubated with QDs for 24 and 48 h. QD untreated cells were used as a control, and the viability was defined as 100%. After incubation, 10 µL of CCK-8 reagent was added to each well and incubated for 2 h at 37 °C in 5% CO_2_ at 90% humidity. The change in the medium colour corresponds to the amount of dye produced in the sample and is directly proportional to the number of viable cells. The optical density was measured using a spectrophotometer Bio-Tek *ELx808* (BioTek Instruments, USA) at a wavelength of 450 nm. The background signal of QDs from all of the tested concentrations was subtracted from the respective samples. Data were analysed in Microsoft Excel and GraphPad Prism software.

### QD release assay

A total of 1 × 10^5^ MSCs were first labelled with 16 nM QDs for 6 h in complete medium. After the primary labelling, the cell-culture supernatant was aspirated, the cells were rigorously rinsed and fresh complete or serum-free medium was added. The number of QD-positive cells was assessed using flow cytometry after 24 and 48 h. The supernatant of the primarily QD-labelled cells was collected at 24 and 48 h and subsequently applied to unlabelled cells for secondary labelling. After 24 h of incubation, the secondarily labelled cells were analysed using flow cytometry to evaluate the uptake of QD. To analyse the effect of proliferation on QD loss from the cells, QD labelled MSCs were propagated either in complete or serum-free medium and assessed for Ki67 expression (as described in method “MSC proliferation assay”) and QD signal using flow cytometry.

### MSC proliferation assay

The effect of QD accumulation on the proliferation of MSCs was evaluated after 24 and 48 h of incubation using the FITC Mouse Anti-Ki67 Set according to the manufacturer’s instructions (BD Bioscience, USA). MSCs were seeded at a density of 5 × 10^4^ cells per well onto 12-well plates in complete medium and allowed to adhere overnight. The medium was subsequently aspirated, and the wells were rinsed once with serum-free medium. The cells were serum-starved for 24 h to synchronize the cell cycle. Next, 16 nM of QDs in complete medium were added, and the cells were incubated for 24 or 48 h. Control wells contained cells in complete medium only. Subsequently, the cells were harvested by trypsinization, washed in PBS and centrifuged for 5 min at 250*g*. The cell pellet was fixed by suspending in 1 mL of 70% ice-cold ethanol. The samples were incubated at −20 °C for at least 2 h. The cells were subsequently washed twice with 9 mL of 1% FBS in PBS at 250*g* for 7 min. Cell pellets were resuspended in 100 μL of PBS, and 10 µL of FITC mouse anti-Ki-67 antibody and isotype control IgG1-FITC were added to the cell suspension, mixed gently and incubated at room temperature for 30 min in the dark. After incubation, the cells were washed with 1 mL of PBS and centrifuged for 5 min at 300*g*. The pellet was suspended in 200 µL of PBS. Nonlabelled cells were used as a control to set the base line of Ki67 expression in MSCs. The isotype control was used to set the Ki67 negative population. The samples were analysed in channel FL-1 using flow cytometry.

### Mesenchymal stem cell tri-lineage differentiation

MSCs were cultivated in complete medium up to 80% confluence. Differentiation into osteogenic, adipogenic and chondrogenic lineages was performed using StemPro Adipogenesis, Chondrogenesis, and Osteogenesis kits according to the manufacturer’s instructions (all from ThermoFisher Scientific, USA). Briefly, for osteogenic differentiation, cells were seeded at a density of 1 × 10^4^/cm^2^ onto 24-well plates. Osteogenic differentiation medium was added; the medium was changed every three days over a period of 21 days. Spontaneous osteodifferentiation control samples were propagated in complete medium for 21 days. Adipogenic differentiation was performed after cultivating 1.82 × 10^4^ cells in 24-well plates using adipogenic differentiation medium. The medium was changed every three days for 21 days. Spontaneous adipodifferentiation control samples were propagated in complete medium for 21 days. For the chondrogenic differentiation assay, 5 µL of a cell suspension with a density of 1.6 × 10^7^ cells/mL in complete medium was seeded onto 96-well plates and incubated for 2 h under high-humidity conditions at 37 °C and 5% CO_2_. Chondrogenic differentiation medium was added, and the medium was changed every three days for 14 days. Spontaneous chondrodifferentiation control samples were propagated in complete medium for 14 days.

Samples were incubated with 8 nM QDs in complete medium for 3 h before starting the differentiation assay. The QD concentration and incubation time were adjusted for the differentiation assay. After incubation with QDs, the medium was discarded, cells were washed with PBS and the relevant differentiation medium was added.

### Evaluation of mesenchymal differentiation

Osteogenic differentiation was evaluated using Alizarin Red S staining. The cells were washed with 1 mL of PBS and fixed with 4% paraformaldehyde (PFA) at room temperature for 30 min. After fixation, the cells were washed two times with distilled water and stained with a 2% Alizarin Red S solution in water (pH adjusted to 4.2 with a 0.1% solution of NH_4_OH) for 45 min at room temperature in the dark. Then, the stained cells were washed four times with 1 mL of distilled water and imaged using EVOS XL microscope (Invitrogen, USA). Samples stained with Alizarin Red S were extracted for quantitative measurements of osteogenic differentiation using 300 µL of 5% perchloric acid and gentle agitation for 10 min at room temperature. Subsequently 100 µL was transferred to a 96-well plate, and the absorbance was measured at 425 nm using an Infinite 200 PRO plate reader and i-control software (Tecan Trading AG, Switzerland).

Adipogenic differentiation was evaluated using Oil Red O staining. Cells were washed with PBS and fixed with 4% formaldehyde for 30 min at room temperature. After fixation, cells were washed with distilled water. Prior to staining, cells were incubated for 5 min at room temperature with 60% isopropanol and subsequently stained with 180 mg/L Oil Red O solution in isopropanol/water (3:2, v/v) for 15 min at room temperature. After staining, the cells were washed four to five times with distilled water and imaged.

Chondrogenic differentiation was evaluated using Alcian Blue staining. Cells were washed once with PBS and fixed with 4% PFA for 30 min at room temperature. After fixation, cells were washed with PBS and stained with a 1% Alcian Blue staining solution in 0.1 M HCl overnight at room temperature. Stained cells were washed three times with 0.1 M HCl and imaged in water.

Quantification of the Alcian Blue stain was achieved by solubilizing the stain in 50 µL of 6 M guanidine hydrochloride (Sigma-Aldrich, USA) overnight at room temperature. Absorbance was measured at 620 nm directly in a 96-well plate using an Infinite 200 PRO plate reader and i-control software.

### Confocal microscopy

For confocal microscopy analysis, 1 × 10^4^ cells per well were seeded on 8-well chamber slides (Nunc, Sigma-Aldrich, USA) in complete medium and left to adhere overnight at 37 °C, 5% CO2 and more than 90% humidity. 16 nM QDs diluted in complete medium were added, and samples were incubated from 15 min to 24 h. Control wells contained nonlabelled cells. After incubation, the medium was aspirated and each well was rinsed with 2 mL of PBS. Then, fixation with 4% PFA in PBS (w/v) for 20 min at room temperature was performed. Wells were washed three times with 0.5 mL of washing buffer containing 1% BSA (Sigma-Aldrich, USA) in PBS for 5 min each. Permeabilization and blocking was performed with 0.3% Triton X-100 (Sigma-Aldrich, USA) and 1% BSA in PBS for 45 min at room temperature. The cytoskeleton of cells was subsequently stained with methanolic Alexa Fluor488 Phalloidin (Thermo Fisher Scientific, USA) diluted 1:100 in washing buffer and incubated for 30 min at room temperature in the dark. The samples were subsequently washed three times and counterstained with a Hoechst 33342 trihydrochloride, trihydrate (10 mg/mL) solution (Thermo Fisher Scientific, USA) diluted 1:1000 in washing buffer for 5 min at room temperature in the dark. Samples were rinsed once with PBS, mounted with ProLong Gold anti-fade mounting medium (Thermo Fisher Scientific, USA) and incubated overnight at room temperature in the dark. Samples were analysed using a Nikon eclipse Ti microscope equipped with a Nikon C2 confocal system. A Nikon S Plan Fluor ELWD 40×/0.60 objective was used. For Alexa Fluor488 Phalloidin, 488 nm was used for excitation, but for Hoechst and QD655, 405 nm lasers were used for excitation. To detect fluorescence for Hoechst - 447/60 nm, Alexa Fluor488 Phalloidin - 525/50 nm and QD655 - 561 LP band pass filters were used (Nikon, Japan). Each channel was recorded separately to avoid spectral overlap. The images were analysed using Nis-Elements C 4.13 software (Nikon, Japan).

### Endocytosis inhibitor assay

To analyse the pathway of QD uptake in MSCs, five endocytosis inhibitors were selected: the clathrin pathway inhibitor chlorpromazine (CPZ), phagocytosis inhibitor cytohalasin D (CytD), macropinocytosis inhibitor ethylisopropyl amiloride (EIPA) (Cayman Chemical, USA), caveolin/lipid raft-mediated endocytosis inhibitor nystatin and caveolin-dependent endocytosis inhibitor dynasore (all from Sigma-Aldrich, USA, unless otherwise stated). The optimal inhibitor concentration was selected using the CCK-8 viability assay. Briefly, 5 × 10^3^ cells per well were seeded on a 96-well plate in 100 μL of complete medium. The next day, endocytosis inhibitors were added in serial dilutions with a twofold dilution factor. The range of the tested inhibitor concentrations was from 1.25–160 μM. The cells were incubated with inhibitors for 24 h. After incubation, 10 µL of CCK-8 reagent was added to each well and incubated for 2 h at 37 °C in 5% CO_2_ at 90% humidity. The optical density was recorded on a Bio-Tek *ELx808* instrument at 450 nm (BioTek Instruments, USA).

MSCs were seeded onto 8-well chamber slides with 2 × 10^4^ cells per well in 0.5 mL of complete medium and incubated for 1 h with the respective inhibitors at the following concentrations: 40 µM CPZ, 2 µM CytD, 5 µM EIPA, 80 µM nystatin and 80 µM dynasore, at 37 °C, 5% CO_2_ and 95% humidity. The medium was aspirated from the wells, and 16 nM QDs were added to samples in complete or serum-free medium and incubated for 6 h. The medium was aspirated and samples were rinsed with 2 mL of PBS. Control wells contained nonlabelled cells. The samples were subsequently stained with methanolic Phalloidin Alexa Fluor488 (Thermo Fisher Scientific, USA) as previously described and analysed using confocal microscopy.

Quantification of the QD fluorescent signal was achieved using Nis-Elements C 4.13 software. Single cell borders were defined according to the Phalloidin Alexa488 staining. The mean fluorescence was measured in the middle z-section of the cell in the red channel only. As a control, the background mean fluorescence from different parts of the image was measured. The QD fluorescence intensity of single cells was calculated by subtracting the background mean intensity from the single-cell mean intensity average.

### Transfection assay

Analogous to the description in [61], transient transfection of MSCs was performed using Cell Light^®^ Reagent-GFP, BacMam 2.0 (Thermo Fisher Scientific, USA) according to the manufacturer’s recommendations. Briefly, MSCs were seeded at a density of 1.5 × 10^4^ cells per well onto 12-well plates in complete growth medium. After the cells attached, BacMam 2.0 reagent was added at a concentration of 80 particles per cell (PPC). Cell Light^®^ Early endosomes-GFP, BacMam 2.0 was used to label early endosomes (Rab5a-GFP expression), Cell Light^®^ Late endosomes-GFP, BacMam 2.0 was used to label late endosomes (Rab7a-GFP expression), and Cell Light^®^ Lysosomes-GFP, and BacMam 2.0 was used to label lysosomes (Lamp1-GFP expression). The cells were transfected for 72 h.

### QD localization study

Transfected MSCs were trypsinized and seeded onto 8-well chambered coverslips (Nunc, Thermo Fisher Scientific, USA) at a density of 3 × 10^4^ cells per well in medium to adhere overnight, and 16 nM of QDs diluted in complete growth medium were added, followed by incubation for 30 min and 1, 6, 24 and 48 h. After incubation, the medium was aspirated and each well was rinsed with PBS. To label nuclei, Hoechst 33342 (Sigma-Aldrich) was diluted in a complete growth medium to a concentration of 25 µg/mL and added to the wells, and the cells were immediately imaged with a laser scanning confocal microscope (Nikon Eclipse TE2000-S, C1 Plus (Nikon, Japan)) using an oil-immersion 60× NA1.4 objective (Plan Apo VC (Nikon, Japan)). A diode laser (404 nm) was used for Hoechst, an argon ion laser (488 nm) for GFP, and a helium–neon laser (543 nm) for QDs. The images were captured with the EZ-C1 v3.90 image analysis software (Nikon, Japan) and further processed using EZ-C1 Bronze v3.80 (Nikon, Japan) and ImageJ 1.48 (National Institute of Health, USA) software.

### Statistical analysis

Statistical analysis was performed using GraphPad Prism Software (Graph Pad Inc., USA). The data are expressed as the representative results or the means of at least three independent experiments +/- standard error of the mean. Statistical significance was analysed using one-way ANOVA. Significance was represented as *p-value < 0.05, **p-value < 0.01, ***p-value < 0.001.
